# Supplementary material for: Secondary Metabolites from Coral-Associated Fungi: Source, Chemistry and Bioactivities
Source: J Fungi (Basel). 2022 Oct 3;8(10):1043. doi: 10.3390/jof8101043 (PMC9604832; doi:10.3390/jof8101043)
Supplement: Supplementary file 1 [file jof-08-01043-s001.zip › jof-1915514-supplementary.pdf]

Journal of Fungi

MDPI

SUPPLEMENTARY MATERIAL

Secondary Metabolites from Coral-associated Fungi: Source, Chemistry and Bioactivities

Ying Chen [a,b], Xiaoyan Pang [a], Yanchun He [a,b], Xiuping Lin [a], Xuefeng Zhou [a], Yonghong Liu [a,*] and Bin Yang [a,*]

1 CAS Key Laboratory of Tropical Marine Bio-resources and Ecology/Guangdong Key Laboratory of Marine Materia Medica, South China Sea Institute of Oceanology, Chinese Academy of Sciences, Guangzhou 510301, China

2 University of Chinese Academy of Sciences, 19 Yuquan Road, Beijing 100049, China

* Correspondence: yonghongliu@scsio.ac.cn (Y.L.); yangbin@scsio.ac.cn (B.Y.)

Abstract: Research efforts of the secondary metabolites of coral-associated fungi lead to a valuable but extra-large chemical database. Many of them exhibit strong biological activity and can be used for promising drug lead compounds. Serving as an epitome of the most promising compounds, which take the ultra-new skeletons and/or remarkable bioactivities, this review presents an overview of new compounds and bioactive compounds isolated from coral-associated fungi, covering the literature from 2010 to 2021. Its emphasis included 423 metabolites, focusing on bioactivity and structure diversity of these compounds. According to structures, these compounds can be roughly classified as terpenes, alkaloids, peptides, aromatics, lactones, steroids and other compounds. Some of them described in this review possess a wide range of bioactivities, such as anticancer, antimicrobial, antifouling and other activities. This review aims to provide some significant chemical and/or biological enlightenment for the study of marine natural products and marine drug development in the future.

Keywords: coral-derived fungus; biological activities; natural products.

Content

Table S1. Anticancer activity 3
Table S2. Antimicrobial activities 5
Table S3. Anti-inflammatory activities.. 8
Table S4. Antifouling activities 9
Table S5. Other activities.. 10
Table S6. Distribution of the compounds according to chemical structure. 13
Table S7. The strain source of the natural products from coral-derived fungi. 13

Table S1. Anticancer activity

Anticancer activity				
Name	Ref.	cell type	IC_{50}	Origin
hirsutanol A (**8**)	[1]	SW620, SW480, LoVo, Hep3B, HepG2, Bel-7402, A549, CNE1, CNE2, SUNE1, MCF7, MDA-MB-231, MDA-MB-435, MDA-MB-453, HeLa	0.58 to 8.27 μg/ml	*Chondrostereum* sp.
incarnal (**7**)	[2]	CNE1, CNE2, SUNE, lovo, KB, Be17402, MCF-7	2.16 to 28.55 μg/ml	*Chondrostereum* sp.
chondrosterin A (**1**) *	[3]	A549, CNE2, and LoVo	2.45, 4.95, 5.47 μM	*Chondrostereum* sp.
(+)-sydowic acid (**32**)	[4]	murine leukemia P-388 cells	2.56 μg/mL	*Aspergillus versicolor*
1-hydroxyboivinianic acid (**34**) *	[5]	A549, Caski, HepG 2 and MCF-7	90.6, 78.2, 75.8, 80.4 μg/mL	*Scopulariopsis* sp.
neoechinulin A (**40**)	[6]	Hela cells	-	*Nigrospora oryzae*
tardioxopiperazine A (**43**)	[7]	NCI-H1975/GR cell lines	50.94%, μM/L	*Nigrospora oryzae*
variecolorin L (**44**)	[7]	NCI-H1975/GR cell lines	56.83%, 50 μM/L	*Nigrospora oryzae*
L-alanyl-L-tryptophan anhydride (**46**)	[8]	TMEM16A	65.0%, 5 mu g/mL	*Nigrospora oryzae*
(-)-variecolorin G (**57**)	[7]	NCI-H1975/GR cell lines	40.65%, 50 μM/L	*Aspergillus* sp.
notoamide G (**62**)	[9]	hepatocellular carcinoma (HCC)	0.66 ± 0.55 to 2.70 ± 0.12 μM	*Aspergillus ochraceus*
avrainvillamide (**63**)	[9]	hepatocellular carcinoma (HCC)	0.63 ± 0.55 to 3.39 ± 0.15 μM	*Aspergillus ochraceus*
stephacidin B (**64**)	[9]	hepatocellular carcinoma (HCC)	0.42 ± 0.55 to 3.19 ± 0.24 μM	*Aspergillus ochraceus*
versiquinazolines L–Q (**74–79**) *	[10]	A549	IC_{50} > 10 μM	*Aspergillus versicolor*
chaetoglobosin E (**95**)	[11-13]	HCT-116, K562, A549, Huh7, H1975, MCF-7, U937, BGC823, HL60, Hela, MOLT-4	13.53±1.90, 8.9, 5.9, 1.4, 9.2, 2.1, 1.4, 8.2, 2.5, 2.8, 1.4 μM,	*Chaetomium globosum*
chaetoglobosin Fex (**96**)	[11]	Huh7, MCF-7, U937, and MOLT-4 cell lines	3.0, 7.5, 4.9, and 2.9 μM	*Chaetomium globosum*
aspergillin PZ (**103**)	[14, 15]	A2780, LNCaP and PC3 cell lines		*Aspergillus elegans*
aspochalasin E (**108**)	[16, 17]	B16-F10 and HCT-116	18.5, 6.3 μg/ml	*Aspergillus* sp
(3*R*,6*R*)-bassiatin (**139**)	[18]	MDA-MB-435, Calu3	7.34 ± 0.20, 14.54 ± 0.01 μM	Dichotomomyces sp.
neoaspergillic acid (**134**)	[19, 20]	SPC-A-1, BEL-7402, SGC-7901, K562	22.2, 24.9, 8.2, 8.0	*Aspergillus* sp.
scopularide A (**162**)	[21]	L5178Y	1.2 μM	*Scopulariopsis* sp.
aspersymmetide A (**150**) *	[22]	NCI-H292 and A431	53.8%, 63.62% (10μM)	*Carijoa* sp.
cladospolide E (**202**) *	[23]	lipid-lowering activity in HepG2 hepatocytes	12.1 μM	*Cladosporium* sp.
secopatulolide A (**204**)	[23]	lipid-lowering activity in HepG2 hepatocytes	8.4 μM	*Cladosporium* sp.
secopatulolide C (**205**)	[23]	lipid-lowering activity in HepG2 hepatocytes	13.1 μM	*Cladosporium* sp.
11-hydroxy- γ -dodecalactone (**206**)	[23]	lipid-lowering activity in HepG2 hepatocytes	7.1 μM	*Cladosporium* sp.

penimethavone A (**252**)	[24]	Hela and rhabdomyosarcoma cell lines	8.41 and 8.18 μM	*Penicillium chrysogenum*
coniochaetone K (**234**) * coniochaetone A (**235**), 8-hydroxy-6-methylxanthone-1-carboxylic acid (**236**), methyl 8-hydroxy-6-methyl-9-oxo-9H-xanthene-1- carboxylate (**237**), methyl 8-hydroxy-6-(hydroxymethyl)-9-oxo-9H-xanthene-1-carboxylate (**238**), 8-(methoxycarbonyl)-1-hydroxy-9-oxo-9H-xanthene-3-carboxylic acid (**239**)	[25]	C4-2B, 22RV1	55.8% to 82.1%, 10 μM	*Cladosporium halotolerans*
coniochaetone A (**235**),	[26]	K562, HL-60, Hela, BGC-823	36.1%, 62.4%, 13.9%,11.4%,100 μg/mL	*Cladosporium halotolerans*
3,8-dihydroxy-6-methyl-9-oxo-9H-xanthene-1-carboxylate (**240**)	[25]	two human prostatic cancer cell lines, C4-2B and 22RV1	82.1%, 77.7%, 10 μM	*Cladosporium halotolerans*
AGI-B4 (**242**)	[21]	cytotoxicity against L5178Y mouse lymphoma cells	1.5 μM	*Scopulariopsis* sp.
altersolanol B (**245**)	[27]	activity against HCT-116 and MCF-7 cancer cell line	3.5 μM, 9.0 μM	*Stemphylium lycopersici*
altersolanol A (**246**)	[27]	HCT-116, MCF-7, Huh7	38.0 μM	*Stemphylium lycopersici*
chrysophanol (**257**)	[28, 29]	against human malignancy of colorectal cancer		*Trichoderma harzianum*
(+)-20-*S*-isorhodoptilometrin (**260**)	[30]	HepG2, HeLa	2.10, 8.59 μM	*Trichoderma harzianum*
1-hydroxy-3-hydroxymethylanthraquinone (**261**)	[30]	hepatoma cell line HepG2	9.39 μM	*Trichoderma harzianum*
nidurufin (**263**)	[31-33]	K562 and HL-60	0.87, 1.46 μM	*Asperigillus* sp.
secalonic acid D (**268**)	[34-36]	K562 cell cycle, pancreatic carcinoma PANC-1 cells	0.6 μM.	*Penicillium* sp.
alterporriol P (**274**) *	[37]	PC-3 and HCT-116	6.4, 8.6 μM	*Alternaria* sp.
alternatone A (**289**) *	[39, 40]	hepatoma carcinoma HepG-2 cell line		*Alternaria alternata*
alterperylenol (**290**)	[39]	A-549, HCT-116, and HeLa cell lines	2.6, 2.4, 3.1 μM	*Alternaria alternata*
verruculosin A (**291**) *	[41]	CDC25B	0.38 ± 0.03 μM	*Talaromyces verruculosus*
bacillisporin F (**293**) *	[41]	CDC25B	0.40 ± 0.02μM	*Talaromyces verruculosus*
xenoclauxin (**294**)	[41]	CDC25B	0.26 ± 0.06μM	*Talaromyces verruculosus*
(–)-bis-dechlorogeodin (**324**)	[42]	Jurkat, A549, and HeLa	10.69, 10.69, 3.56 μM	*Aspergillus* sp.
penicitol I (**307**) *	[43]	A549, BEL-7402	19, 17 μM	*Penicillium citrinum*
tenellic acid A methyl ester (**322**)	[44]	HepG2, Hep3B, MCF-7/ADR, PC-3, HCT-116	4.3±0.3, 9.0±0.5, 8.2±0.7, 9.8±0.9 μM	*Talaromyces* sp
geodin (**328**)	[45]	BT474, NCI-H460, H-1975, K562, DU145, A549	8.88, 9.22, 9.96, 11.14, 14.44, 11.05 μM	*Sinularia* sp.
violaceol I (**329**)	[21]	L5178Y	9.5μM	*Stylophora* sp.
violaceol II (**330**)	[21]	L5178Y	9.2 μM	*Stylophora* sp.

butyrolactone I (**344**)	[46-48].	HL-60	13.22 μM	*Aspergillus terreus*.
satratoxin F (**364**)	[49]	MDA-MB-231, C4-2B, MGC803, MDA-MB-468, A549	< 39 nM	*Stachybotrys chartarum*
stachybotrylactone B (**394**)	[50]	HL-60, K562, MOLT-4, ACHN, 786-O, OS-RC-2	5.23, 4.12, 4.31, 23.55, 7.65, 10.81 μmol/L	*Penicillium* sp.
purpactin A (**416**)	[51-54]	inhibitor of TMEM16A chloride channels<br>MCF7, H460, SF268	2 μM<br>20.5, 17.6, 21.9 μM	*Penicillium pinophilum*
cladosporilactam A (**423**) *	[55]	Hela	0.76 μM	*Cladosporium* sp.

Table S2. Antimicrobial activities

Antimicrobial activities				
Name	Ref.	Type	Evaluation	Origin
(*R*)-(–)-hydroxysydonic acid (**15**)	[56]	*S. aureus, B. cereus, K. rhizophila, P. putida, P. aeruginosa, S. enterica, N. brasiliensis*	-	*Aspergillus* sp.
(*S*)-(–)-5-(hydroxymethyl)-2-(2',6',6'-trimethyltetrahydro-2*H*-pyran-2-yl) phenol (**16**)	[56]	*S. aureus, B. cereus, K. rhizophila, P. putida, P. aeruginosa, S. enterica, N. brasiliensis*	-	*Aspergillus* sp.
(*S*)-(+)-11-dehydrosydonic acid (**17**)	[56].	*S. aureus, B. cereus, K. rhizophila, P. putida, P. aeruginosa, S. enterica, N. brasiliensis*	-	*Aspergillus* sp.
(+) - methyl sydowate (**27**) *	[57]	*Staphylococcus aureus* and methicillin resistant *S. aureus*	100 μg/mL zones of 11 mm in diameter	*Aspergillus* sp.
7-deoxy-7, 14-didehydrosydonic acid (**28**) *	[58]	*G. graminis*	MIC = 0.5 μg/mL	*Dichotella gemmacea*
(+)-sydonic acid (**30**)	[59]	*S. albus, S. aureus*	MIC = 5.3, 2.6 μg/mL	*Sarcophyton* sp.
expansol G (**31**)	[59]	*S. albus, S. aureus*	MIC = 6.4, 6.4 μg/mL	*Sarcophyton* sp.
(+)-sydowic acid (**32**)	[59]	*S. albus, S. aureus*	MIC = 5.4, 5.4 μg/mL	*Sarcophyton* sp.
1-hydroxyboivinianic acid (**34**) *	[5]	*Erwinia carotovora* sub sp.	MIC = 68.9 μg/mL	*Stylophora* sp.
craterellin A (**36**)	[60]	*Bacillus cereus*	MIC = 3.12 μM	*Lophiostoma* sp.
neoechinulin A (**40**)	[6]	anti-viral	-	*Nigrospora oryzae*
cyclo-(Pro-Val) (**69**)	[61]	inhibition to the MptpB	IC_{50} = 25.9 μM	*Simplicillium* sp.
fumiquinazoline L (**84**)	[62]	*Bacillus subtilis*, *Staphylococcus albus*, and *Vibrio parahemolyticus*	MIC = 50 μM	*Scopulariopsis* sp.
cottoquinazoline D (**82**) *	[63]	antifungal activity against *Candida albicans*	MIC = 22.6 μM	*Aspergillus versicolor* LCJ-5-4
aspochalasin I (**99**)	[14]	*S. epidermidis* and *S. aureus*	MIC = 20, 10 μM	*Sarcophyton* sp.
aspochalasin D (**101**)	[14]	*Staphylococcus albus*, *S. aureus*, *Escherichia coli* and *Bacillus cereus*	MIC = 10 μM	*Sarcophyton* sp.
aspergillin PZ (**103**)	[14]	*S. epidermidis*	MIC = 20 μM	*Sarcophyton* sp.
chaetoglobosin A (**104**)	[64]	*Tetragenococcus halophilus*	MIC = 0.7 μM	*Chaetomium globosum*
chaetoglobosin B (**105**)	[64]	*Tetragenococcus halophilus*	MIC = 0.4 μM	*Chaetomium globosum*

cytoglobosin C (**106**)	[64]	*T. halophilus*	MIC = 0.7 μM	*Chaetomium globosum*
aniduquinolone A (**109**)	[65]	*staphylococcus aureus* (ATCC700699)	-	*Scopulariopsis* sp.
6-deoxyaflaquinolone E (**112**)	[66]	*S. aureus, B. cereus, V. parahaemolyticus, N. brasiliensis, and P. putida*	MIC = 0.78, 1.56, 6.25, 0.78, 1.56 μM,	*Scopulariopsis* sp.
asperteramide A (**132**)	[67]	*C. albicans* antibacterial activity against six drug-resistant microbial pathogens, including *E. coli, A. Baumannii, P. aeruginosa, K. pneumonia*, MRSA, and *E. faecalis*	MIC = 2 μg/mL MIC = 8, 8, 16, 64, 64, 8 μg/ml	*Aspergillus terreus*
4'-OMe-asperphenamate (**173**) *	[14]	*S. epidermidis*	MIC = value of 2 μg/mL	*Sarcophyton* sp.
asperphenamate (**174**) *	[14]	*S. epidermidis*	MIC = 2 μg/mL	*Sarcophyton* sp.
pyrophen (**137**)	[68]	*C. albicans*	zone 28 mm (40 μg/disc)	*Alternaria alternata*
neoaspergillic acid (**134**)	[19, 20]	*S. aureus, S. epidermidis, B. subtilis, B. dysenteriae, B. proteus, E. coli*	MIC = 1.0, 0.5, 1.9, 7.8, 7.8, 15.6 μg/mL	*Aspergillus* sp.
22-O-(N-Me-L-valyl)-21-epi-aflaquinolone B (**131**) *	[69]	anti-RSV activity	IC_{50} = 42 nM	*Aspergillus* sp.
sinulariapeptide A (**158**) *	[61]	*Colletotrichum asianum*	MIC = 4.9 µg/mL	*Simplicillium* sp.
simplicilliumtide A (**159**)	[61]	*Colletotrichum asianum, Pyricularia oryza* Cav	MIC = 9.8, 19.5 µg/mL	*Simplicillium* sp.
simplicilliumtide B (**160**)	[61]	*Colletotrichum asianum, Pyricularia oryza* Cav	MIC = 4.9 9.8 µg/mL	*Simplicillium* sp. SCSIO 41209
simplicilliumtide J (**161**)	[61]	*Colletotrichum asianum, Pyricularia oryza* Cav	MIC = 19.5, 78.1 µg/mL	*Simplicillium* sp. SCSIO 41209
asperpeptide A (**163**) *	[70]	*Bacillus cereus* and *Staphylococcus epidermidis*	MIC = 12.5 μM	*Aspergillus* sp.
hirsutellic acid A (**169**)	[61]	inhibition to the MptpB	IC_{50} = 35.0 μM	*Simplicillium* sp.
(+) - pestaloxazine A (**170**) *	[71]	antiviral activity to EV71	IC_{50} = 14.2 ± 1.3 μM	*Pestalotiopsis* sp.
aspergillipeptide E (**183**) *	[72]	activity against HSV-1	IC_{50} = 19.8 μM	*Aspergillus* sp.
isochromophilone IX (**212**)	[73]	against MRSA	MIC = 50 μg/mL	*Penicillium sclerotiorum*
(+)-sclerotiorin (**222**)	[74, 75]	*B. subtilis*, *B. cereus*, and *S. lutea*	MIC = 0.16, 0.31, 0.31 μM	*Penicillium sclerotiorum*
microketide A (**223**) *	[76]	*Pseudomonas aeruginosa*, *Nocardia brasiliensis*, *Kocuria rhizophila*, and *Bacillus anthraci*	MIC = 0.19 μg/mL	*Microsphaeropsis* sp.
arthproliferin A (**226**) *	[49]	methicillin-resistant *Staphylococcus aureus* ATCC 29213	MIC = 78 μg/mL	*Stachybotrys chartarum*
tetrahydroaltersolanol C (**247**) *	[77]	e PRRSV	IC_{50} = 65 μM	*Alternaria* sp.
frangulaemodin (**258**)	[30]	*S. aureus*	MIC = 6.25 μM	*Trichoderma harzianum*
(+)-20*S*-isorhodoptilometrin (**260**)	[30]	*S. aureus*	MIC = 25.0 μM	*Trichoderma harzianum*
1-hydroxy-3-hydroxymethylanthraquinone (**261**)	[30]	*S. aureus*	MIC value of 25.0 μM	*Trichoderma harzianum*
8-O-methylnidurufin (**266**)	[31-33]	*M. luteus*	MIC = 6.25 μM.	*Asperigillus* sp.
8-O-methylaverufin (**264**)	[31-33]	*M. luteus*<br>*Mucor miehei*	MIC = 6.25 μM.	*Asperigillus* sp.
secalonic acid D (**268**)	[34-36]	against *S. aureus* biofilm formation	>90% at 6.25 μg/mL	*Penicillium* sp.
secalonic acid D (**268**)	[79]	*M. luteus*, *P. nigrifaciens*, *E. coli*, *B. subtilis* inhibition to the growth of *S. onedensis* MR-1	MIC = 24.4, 97.5, 24.4, 24.4 μg/mL	*Penicillium* sp.

			3.125 μg/mL	
secalonic acid B (**269**)	[34-36]	against *S. aureus* biofilm formation	>90% at 6.25 μg/mL	*Penicillium* sp.
secalonic acid B (**269**)	[79]	*M. luteus*, *P. nigrifaciens*, *E. coli*, *B. subtilis*	MIC values of 97.5, 390.5, 97.5, 97.5 μg/mL	*Penicillium* sp.
penicillixanthone A (**270**)	[79]	*M. luteus*, *P. nigrifaciens*, *E. coli*, *B. subtilis*	MIC values of 24.4, 97.5, 24.4, 24.4 μg/mL	*Penicillium* sp. SCSGAF 0023
alterporriol Q (**275**) *	[77]	activity against PRRSV	IC_{50} = 22 μM	*Alternaria* sp.
(±) - pestalachloride D (**281**) *	[80]	*Escherichia coli*, *Vibrio anguillarum* and *Vibrio parahaemolyticus*	MIC= 5.0, 10.0, 20.0 μM	*Pestalotiopsis* sp.
(±) - pestalachloride C (**280**)	[80]	*Escherichia coli*, *Vibrio anguillarum* and *Vibrio parahaemolyticus*	the MIC values of 5.0, 10.0 and 20.0 μM	fungus *Pestalotiopsis* sp.
pestalachloride B (**279**)	[81]	Weak activities against *S. aureus*.	MIC = 25.00, 25.00, 50.00, 25.00, 50.00 μM	*Pestalotiopsis* sp.
stemphyperylenol (**287**)	[82-84]	*e.coli* , *Staphylococcus aureus* and multi-drug resistant *Pseudomonas aeruginosa*	-	*Alternaria* sp.
bacillisporin F (**293**) *	[41, 85].	*S. aureus*	MIC = 15.6±0.3 μg/mL	*Talaromyces verruculosus*
phomalichenone D (**297**)	[86]	MRSA shhs-A1	32–64 μg/mL	*Parengyodontium album* sp.
sulochrin (**323**)	[42]	*V. anguillarum* and *A. salmonicida, P. aeruginosa*	MIC = 15.06/15.15, 7.53 μM	*Aspergillus* sp.
(–)-bis-dechlorogeodin (**324**)	[42]	*V. anguillarum* and *A. salmonicida, P. aeruginosa*	MIC = 30.12/30.30, 3.78 μM	*Aspergillus* sp.
3,7-dihydroxy- 1,9- dimethyl dibenzofuran (**299**)	[87].	against *E. coli*, MRSA, *S. aureus* and *E. faecalis*	MIC = 0.45~ 15.6 μg/mL	*Talaromyces* sp.
phomalichenone A (**300**)	[86]	MRSA shhs-A1 and *M. tuberculosis* H37Ra	MIC = 64/64 μg/mL	*Parengyodontium album* sp.
phomalichenone B (**301**)	[86]	MRSA shhs-A1 and *M. tuberculosis* H37Ra	MIC = 16/64, μg/mL	*Parengyodontium album* sp.
isodihydroauroglaucin (**325**)	[88, 89]	HSV-1	EC_{50} = 4.73 μM	*Aspergillus ruber*
flavoglaucin (**326**)	[88, 89]	HSV-1	EC_{50} = 6.95 μM	*Aspergillus ruber*
penicitol A (**302**) *	[43]	*Staphylococcus aureus*, *Bacillus subtilis*, and Vancomycin resistant *Enterococcus faecalis* 1010798 (VRE)	MIC =16, 32, 32, 64, 32, 32 μM,	*Penicillium citrinum*.
penicitol I (**307**) *	[43]	*Staphylococcus aureus*, *Bacillus subtilis*, and Vancomycin resistant *Enterococcus faecalis* 1010798 (VRE)	MIC =16, 32, 32, 64, 32, 32 μM	*Penicillium citrinum*.
phomaether A (**319**) *	[90]	*S. albus, S. aureus, E. coli, V. parahaemolyticus, V. anguillarum*	MIC = 0.312 to 10.0 μM	*Phoma* sp.
phomaether C (**321**) *	[90]	*S. albus, S. aureus, E. coli, V. parahaemolyticus, V. anguillarum*	MIC = 0.312 to 10.0 μM	*Phoma* sp.
2,4-diphenyldichloroasterric acid (**327**)	[45]	inhibition to *S. aureus*	MIC value of 12.5 μM	*Aspergillus* sp.
violaceol I (**329**)	[21, 91, 92]	antimicrobial activities	MIC <9.765 to 312.5 μg/mL	*Scopulariopsis* sp.
violaceol II (**330**)	[21, 91, 92]	antimicrobial activities	MIC <9.765 to 312.5 μg/mL	*Scopulariopsis* sp.
8-ethyl-5,7-dihydroxy-2-methylchroman-4-one (**334**)	[86]	MRSA shhs-A1 *M. tuberculosis* H37Ra	MIC = 64, 32 μg/mL	*Parengyodontium album* sp.
(±)-trieusol D (**335**)	[86]	MRSA shhs-A1	MIC = 64, 64 μg/mL	*Parengyodontium album* sp.

		M. tuberculosis H37Ra		
(3*Z*,5*S*,6*E*,8*S*,9*S*,10*R*)-8-chloro-5,8,9,10-tetrahydro-5,9-dihydroxy-10-methyl-2H-oxecin-2-one (**336**)	[60]	*B. cereus*	MIC = 3.12 μM.	*Lophiostoma* sp.
2-*O*-Methylbutyrolactone I (**348**)	[93]	*S. aureus, S. epidermidis, B. cereus, V. parahaemolyticus* and *V. anguillarum*	MIC = 1.56 to 12.5 μM	*Aspergillus* sp.
2-*O*-Methylbutyrolactone II (**349**)	[93]	*S. aureus, S. epidermidis, B. cereus, V. parahaemolyticus* and *V. anguillarum*	MIC = 1.56 to 12.5 μM	*Aspergillus* sp.
satratoxin F (**364**)	[49]	methicillin-resistant *Staphylococcus aureus* ATCC 29213	MIC = 39 μg/mL	*Stachybotrys chartarum*
(*R*)-3-hydroxymellein (**377**)	[94]	MRSA	MIC = 100 μg/mL	*Aspergillus* sp.
(3*R*,4*S*)-trans-4-hydroxymellein (**378**)	[94]	*Enterococcus faecalis*	MIC = 100 μg/mL	*Aspergillus* sp.
5α,8α-epidioxy-ergosta-6,22E-dien-3β-ol (**399**)	[95].	*Escherichia coli, Pseudomonas putida,* and *Kocuria rhizophila*	MIC = 3.13, 1.56, 6.25 μM	*Xylaria* sp.
3α-hydroxy-7-ene-6,20-dione (**400**) *	[96]	antiviral activity	IC_{50} = 0.12 mM	*Cladosporium* sp.
eujavanicol A (**403**)	[97, 98]	*Escherichia coli*	MIC = 5.0 μg/mL	*Trichoderma harzianum*
pseurotin A (**409**)	[99-102].	*Bacilus cereus* and *Shigella shiga*	MIC = 64 mu g/mL.	*Pseudallescheria boydii*

Table S3. Anti-inflammatory activities

Anti-inflammatory activities				
Name	Ref.	Type	Evaluation	Origin
lovastatin (**18**)	[46]	against NO production	17.45 μM	*Aspergillus terreus*
neoechinulin A (**40**)	[6]		-	*Nigrospora oryzae*
chaetoglobosin Fex (**96**)	[11-13]		-	*Chaetomium globosum*
luteoride E (**129**) *	[46]		24.64 μM	*Aspergillus terreus*
methyl 3,4,5-trimethoxy-2-(2-(nicotinamido) benzamido) benzoate (**133**)	[46]		5.48 μM	*Aspergillus terreus*
sclerketide B (**209**) *	[103]		3.4±0.5 μM	*Penicillium sclerotiorum*
sclerketide C (**210**) *	[103]		2.7±0.5 μM	*Penicillium sclerotiorum*
α-pyrone derivative sclerketide D (**211**) *	[103]		5.5±0.5 μM	*Penicillium sclerotiorum*
isochromophilone IX (**212**)	[103]		17.6±0.7 μM	*Penicillium sclerotiorum*
sequoiatone B (**213**)	[103]		5.2±0.6 μM	*Penicillium sclerotiorum*
altersolanol A (**246**)	[104]			*Stemphylium lycopersici*
territrem A (**311**)	[46]		IC_{50} = 29.34 μM	*Aspergillus terreus.*
eurothiocin A (**312**) *	[105, 106]		-	*Eurotium rubrum*
3'-isoamylene butyrolactone IV (**342**) *	[107]		25.1%	*Aspergillus terreus*
butyrolactone I (**344**)	[107]		nearly 25.3%	*Aspergillus terreus*
versicolactone B (**345**)	[107]		20 μM	*Aspergillus terreus*
versicolactone G (**346**) *	[46]		IC_{50} = 15.72 μM	*Aspergillus terreus*
14α-hydroxyergosta-4,7,22-triene-3,6-dione (**396**)	[46]		IC_{50} = 26.83 μM	*Aspergillus terreus*
arthriniumsteroid A (**385**) *	[108]		21.4%−44.6%	*Simplicillium lanosoniveum*

arthriniumsteroid B (**386**) *	[108]			*Simplicillium lanosoniveum*
arthriniumsteroid C (**387**) *	[108]			*Simplicillium lanosoniveum*
arthriniumsteroid D (**388**) *	[108]			*Simplicillium lanosoniveum*
eoaspergillic acid (**389**)	[108]			*Simplicillium lanosoniveum*

Table S4. Antifouling activities

Antifouling activities				
Name	Ref.	Antifouling type	Evaluation	Origin
neoechinulin A (**40**)	[109]	barnacle *Balanus amphitrite*	30.0 μg/mL	*Nigrospora oryzae* (ZJ-2008005)
isoechinulin A (**42**)	[109]	barnacle *Balanus amphitrite*	IC_{50} = 5.92 μg/mL	*Nigrospora oryzae* (ZJ-2008005)
dihydroxyisoechinulin A (**45**)	[109]	barnacle *Balanus amphitrite*	50.0 μg/mL	*Nigrospora oryzae* (ZJ-2008005)
cyclo-(Phe-Phe) (**68**)	[110]	cell line Sf9 from the fall armyworm *Spodoptera frugiperd*	0.8 μM	*Lobophytum crissum*
aspochalasin I (**99**)	[14]	barnacle *Balanus Amphitrite*	EC_{50} = 34 μM	*Sarcophyton* sp.
aspochalasin J (**100**)	[14]	barnacle *Balanus Amphitrite*	EC_{50} = 14 μM	*Sarcophyton* sp.
aspochalasins D (**101**)	[14]	barnacle *Balanus Amphitrite*	EC_{50} = 6.2 μM	*Sarcophyton* sp.
aspochalasin H (**102**)	[14]	barnacle *Balanus Amphitrite*	EC_{50} = 37 μM	*Sarcophyton* sp.
scopuquinolone B (**114**) *	[111]	*B. amphitrite* cyprids	EC_{50} = 0.103 μM	*Scopulariopsis* sp.
aniduquinolone A (**109**)	[66]	*B. amphitrite*	EC_{50} = 17.5 pM	*Scopulariopsis* sp.
aflaquinolone A (**110**)	[66]	B. amphitrite	EC_{50} = 28 nM	*Scopulariopsis* sp.
aflaquinolone D (**111**)	[66]	*B. amphitrite*	EC_{50} = 2.8 nM	*Scopulariopsis* sp.
6-deoxyaflaquinolone E (**112**)	[66]	*B. amphitrite*	EC_{50} = 1.04 μM	*Scopulariopsis* sp.
aflaquinolone F (**113**)	[66]	*B. amphitrite*	EC_{50} = 0.86 μM	*Scopulariopsis* sp.
sinuxylamide A (**115**) *	[112]	fibrinogen to purified integrin IIIb/IIa	IC_{50} = 0.89 μM	*Xylaria* sp.
sinuxylamide B (**116**) *	[112]	fibrinogen to purified integrin IIIb/IIa	IC_{50} = 0.61 μM	*Xylaria* sp.
speradine C (**121**)	[110]	against the Sf9 cells	IC_{50} = 0.9 μM	*Lobophytum crissum*
24,25-dehydro-10,11-dihydro-20-hydro-xyaflavinin (**122**)	[110]	against the Sf9 cells	IC_{50} = 0.5 μM	*Lobophytum crissum*
aflavinine (**123**)	[110]	against the Sf9 cells	IC_{50} = 0.4 μM	*Lobophytum crissum*
aspergilone A (**218**) *	[37]	against *Balanus Amphitrite*	EC_{50} = 25 μg/mL	*Aspergillus* sp.
(+)-sclerotiorin (**222**)	[74, 75]	barnacle *Balanus amphitrite*	EC_{50} = 5.6 µg/mL	*Penicillium* sclerotiorum
6,8,5'6'-tetrahydroxy-30 methyl-flavone (**230**) *	[79]	*Balanus amphitrite* larvae settlement,	6.7 μg/ml	*Penicillium* sp.
emodin (**231**)	[79]	*Balanus amphitrite* larvae settlement,	6.1 μg/ml	*Penicillium* sp.
citreorosein (**232**)	[79]	*Balanus amphitrite* larvae settlement,	17.9 μg/ml	*Penicillium* sp.

isorhodoptilometrin (**233**)	[79]	*Balanus amphitrite* larvae settlement,	13.7 μg/ml	*Penicillium* sp.
altertoxin I (**285**) *	[113]	barnacle *Balanusamphitrite*	IC_{50} = 0.27 μg/mL	*Alternaria* sp.
3,7-dihydroxy- 1,9- dimethyl dibenzofuran (**299**)	[87]	against *Bugula neritina larva*	LC_{50} = 3.06 μg/mL	*Talaromyces* sp.
(l7*R*)-17-methylincisterol (**339**)	[87]	*Bugula neritina* larva	LC_{50} value of 4.15 μg/mL,	*Talaromyces* sp. SCSIO 041201
talaromycin C (**318**) *	[44]	barnacle *Balanus Amphitrite*	EC_{50} = 2.8±0.2 μg/mL	*Talaromyces* sp.
dimethyl incisterol A3 (**338**)	[87]	*Bugula neritina* larva	LC_{50} = 3.13 μg/mL,	*Talaromyces* sp.
2-O-Methylbutyrolactone II (**349**)	[93]	barnacle *Balanus amphitrite*	EC_{50} = 2.10±0.20 μg/mL	*Aspergillus* sp.
demethoxycarbonylbutyrolactone II (**350**)	[93]	barnacle *Balanus amphitrite*	EC_{50} = 4.25±0.32,μg/mL	*Aspergillus* sp.
butyrolactone III (**351**)	[93]	barnacle *Balanus amphitrite*	EC_{50} = 2.98±0.22,μg/mL	*Aspergillus* sp.
cochliomycin A (**352**) *	[114, 115]	barnacle *Balanus Amphitrite*	EC_{50} values of 1.2 μg/mL	*Cochliobolus lunatus*
diacetyl derivative (**355**)	[114, 115]	barnacle *Balanus Amphitrite*	EC_{50} values of 15.4 μg/mL	*Cochliobolus lunatus*
monoacetyl derivative (**356**)	[114, 115]	barnacle *Balanus Amphitrite*	EC_{50} values of 12.5 μg/mL	*Cochliobolus lunatus*
zeaenol (**357**)	[114, 115]	barnacle *Balanus Amphitrite*	EC_{50} values of 5.0 μg/mL	*Cochliobolus lunatus*
LL-Z1640-1 (**358**)	[114, 115]	barnacle *Balanus Amphitrite*	EC_{50} values of 5.3 μg/mL	*Cochliobolus lunatus*
paecilomycin F (**360**)	[114, 115]	barnacle *Balanus Amphitrite*	EC_{50} values of 17.9 μg/mL	*Cochliobolus lunatus*
aspergillide C (**363**) *	[116-118]	*Bugula neritina*	LC_{50}/EC_{50} > 25	*Aspergillus* sp.
(+)-eurotiumide B (**380**) *	[119]	barnacle *Balanus amphitrite*	EC_{50} = 0.7 to 2.3 μg/mL	*Eurotium* sp.
(-)-eurotiumides D (**382**) *	[119]	barnacle *Balanus amphitrite*	EC_{50} = 0.7 to 2.3 μg/mL	*Eurotium* sp.
24,28-didehydro-2 (**398**) *	[93]	barnacle *Balanus amphitrite*	EC_{50} = 18.40±2.0 μg/mL	*Aspergillus* sp.
chaxine C (**397**)	[93]	barnacle *Balanus amphitrite*	EC_{50} = 2.50±0.22 μg/mL	*Aspergillus* sp.
methyl-trichoharzin (**401**) *	[97]	*Bugula neritina*	EC_{50} = 29.8, μg/mL	*Trichoderma harzianum*
eujavanicol A (**403**)	[97]	*Bugula neritina*	EC_{50} = 35.6 μg/mL	*Trichoderma harzianum*
nafuredin (**404**)	[97]	*Bugula neritina*	EC_{50} = 21.4 μg/mL	*Trichoderma harzianum*
hydroxypenicillide (**413**) *	[51-54]	barnacle *Balanus Amphitrite*	EC_{50} = 6.0 μg/mL	*Penicillium pinophilum*
penicillide (**414**)	[51-54]	barnacle *Balanus Amphitrite*	EC_{50} = 2.6 μg/mL	*Penicillium pinophilum*
isopenicillide (**415**)	[51-54]	barnacle *Balanus Amphitrite*	EC_{50} = 20 μg/mL	*Penicillium pinophilum*
purpactin A (**416**)	[51-54]	barnacle *Balanus Amphitrite*	EC_{50} = 10 μg/mL	*Penicillium pinophilum*

Table S5. Other activities

Other activities				
name	reference	Type of bioactivity	evaluation	Origin
chondrosterin B (**2**)	[120]	antimalarial activity	IC_{50} = 3.10μg/mL	*Chondrostereum* sp.
lovastatin (**18**)	[121]	prevention and treatment of neurological disorders; lipid-lowering drug	-	*Aspergillus terreus*
harziane lactone A (**19**) *, harziane lactone B (**20**) *, Harzianones A–C (**21**–**23**) *, harziane (**25**) *	[122]	against amaranth and lettuce	200 ppm	*Trichoderma harzianum*
neoechinulin A (**40**)	[123, 124]	DPPH scavenging effect	IC_{50} = 44.30±0.06 μM	*Nigrospora oryzae*
isoechinulin A (**42**)	[123, 124]	DPPH scavenging effect		*Nigrospora oryzae*
tardioxopiperazine A (**43**)	[125]	Con A-induced & LPS-induced immunosuppressive	IC_{50} = 4.5&0.7 μM	*Nigrospora oryzae*

16,17-dihydroxy-deoxydihydroisoaustamide (**50**) *	[126]	neuroprotective activity	38.6%, 1 μM	*Penicillium dimorphosporum*
16β,17α-dihydroxy-deoxydihydroisoaustamide (**51**) *	[126]	neuroprotective activity	36.5%, 1 μM	*Penicillium dimorphosporum*
16α,17α-dihydroxy-deoxydihydroisoaustamide (**52**) *	[126]	neuroprotective activity	30.3%, 1 μM	*Penicillium dimorphosporum*
avrainvillamide (**63**)	[127, 128]	human GR activity in LNCaP cell lysate; threat to human health in buildings	IC_{50} = 125 μM	*Aspergillus ochraceus*
stephacidin B (**64**)	[127, 128]	threat to human health in buildings	-	*Aspergillus ochraceus*
versiquinazoline P (**78**) *	[10]	thioredoxin reductase	13.6 ± 0.6 μM	*Aspergillus versicolor*
versiquinazoline Q (**79**) *	[10]	thioredoxin reductase	12.2 ± 0.7 μM	*Aspergillus versicolor*
versiquinazolines A, B, G & K (**70-73**)	[129]	TrxR inhibitory activity	IC_{50} = 20±1, 12±2, 13±0, 13±0 μM	*Aspergillus versicolor*
aspochalasin I (**99**)	[130]	inhibition to melanogenesis in Mel-Ab cells	IC_{50} = 22.4 mμ M	*Aspergillus elegans*
aspergillin PZ (**103**)	[14, 15]	DPPH free radical scavenging effects		*Aspergillus elegans*
aflaquinolone A (**110**)	[131]	against brine shrimp	LD_{50} = 5.5 μM	*Scopulariopsis* sp.
neoaspergillic acid (**134**)	[19, 20]	against the brine shrimp	LC_{50} = 90.08 μM	*Aspergillus* sp.
chrysogeamide A (**151**) *	[132]	zebrafish embryo	1.0 μg/mL	*Penicillium chrysogenum*
chrysogeamide B (**152**) *	[132]	zebrafish embryo	1.0 μg/mL	*Penicillium chrysogenum*
versicotides D–F (**147–149**) *	[133]	anti-atherosclerosis activity		*Aspergillus versicolor*
aspergilliamide (**175**) *	[19, 134, 135]	against the brine shrimp	LC_{50} = 71.09 μM	*Aspergillus* sp.
ochratoxin A n-butyl ester (**176**)	[19, 134, 135]	against the brine shrimp	LC_{50} = 4.14 μM	*Aspergillus* sp.
flavacol (**177**)	[19, 134, 135]	against the brine shrimp	LC_{50} =205.67 μM	*Aspergillus* sp.
flavacol (**177**)	[19, 134, 135]	against NADH oxidase	IC_{50} = 13.0±0.4 μM	*Aspergillus* sp.
ochratoxin A (**178**)	[19, 134, 135]	against the brine shrimp	LC_{50} = 13.74 μM	*Aspergillus* sp.
ochratoxin A (**178**)	[19, 134, 135]	potential nephrotoxin and a latent human carcinogen		*Aspergillus* sp.
ochratoxin A methyl ester (**176**)	[19, 134, 135]	against the brine shrimp	LC_{50} = 2.59 μM	*Aspergillus* sp.
libertalide B (**187**) * libertalide H (**193**) * aspermytin A (**200**)	[136]	induced the proliferation of $CD3^+$ T cells.	-	*Libertasomyces* sp.
libertalide E (**208**) *, libertalide I (**194**) *, libertalide M (**197**) *, aspermytin A acetate (**201**)	[136]	increased the $CD4^+/CD8^+$	3 μM	*Libertasomyces* sp.
aspermytin A (**200**)	[137]	against neurite outgrowth	50 μM	*Libertasomyces* sp.
austalide V (**216**)	[138]	against pancreatic lipase	IC_{50} = 23.9 μg/mL	*Penicillium glabrum*
asteltoxin B (**217**) *	[118, 139]	inhibition to human acetylcholinesterase	IC_{50} = 14.9 μM	*Aspergillus* sp.
aspergivone B (**253**) *	[140]	against alpha-glucosidase	IC_{50} = 244 μg/mL	*Aspergillus candidus*
pachybasin (**256**)	[30]	AChE inhibitory activity	100 μM	*Trichoderma harzianum*

pachybasin (**256**)	[28, 29]	toxicity in zebrafish embroys	-	*Trichoderma harzianum*
chrysophanol (**257**)	[30]	AChE inhibitory activity	100 μM	*Trichoderma harzianum*
phomarin (**259**)	[30]	AChE inhibitory activity	100 μM	*Trichoderma harzianum*
(+)-20*S*-isorhodoptilometrin (**260**)	[30]	AChE inhibitory activity	100 μM	*Trichoderma harzianum*
Ω-hydroxydigitoemodin (**262**)	[30]	AChE inhibitory activity	100 μM	*Trichoderma harzianum*
averufanin (**265**)	[31-33]	inhibitory effects on ACAT1 and ACAT2	IC_{50} = 28±1.1, 12±0.1μM	*Asperigillus* sp.
altertoxin I (**285**)	[113]	zebrafish embryo on *Daniorerio*	LC_{50} = 4.54 μg/mL	*Alternaria* sp.
flavoglaucin (**326**)	[88, 89]	(DPPH)	IC_{50} value of 11.3 μM	*Aspergillus ruber*
eurothiocin A (**312**) *	[105]	against α-glucosidase	IC_{50} = 17.1 ± 0.7 μM	*Eurotium rubrum*
eurothiocin B (**313**) *	[105]	against α-glucosidase	IC_{50} = 42.6 ± 1.4 μM	*Eurotium rubrum*
LL-Z1640-2 (**359**)	[114, 115]	$HgCl_2$-induced JNK phosphorylation	5–100 ng/mL	*Cochliobolus lunatus*
6-(5-carboxy-3-methylpent-2-enyl)-7-hydroxy-3,5-dimethoxy-4-methylphthalan-1-one (**365**) *	[141]	inhibition to IMPDH2	IC_{50} = 0.84±0.11μM	*Penicillium bialowiezense*
6-(5-methoxycarbonyl-3-methylpent-2-enyl)-3,7-dihydroxy-5-methoxy-4-methylphthalan-1-one (**366**) *	[141]	inhibition to IMPDH2	IC_{50} = 3.27±0.18μM	*Penicillium bialowiezense*
6-(3-carboxybutyl)-7-hydroxy-5-methoxy-4-methylphthalan-1-one (**367**) *	[141]	inhibition to IMPDH2	IC_{50} = 24.68±2.74μM	*Penicillium bialowiezense*
6-[5-(2,3-dihydroxy-1-carboxyglyceride)-3-methylpent-2-enyl]-7-hydroxy-5-methoxy-4-methylphthalan-1-one (**368**) *	[141]	inhibition to IMPDH2	IC_{50} = 8.59±0.43μM	*Penicillium bialowiezense*
6-[5-(1-carboxy-4-N-carboxylate)-3-methylpent-2-enyl]-7-hydroxy-5-methoxy-4-methylphthalan-1-one (**369**) *	[141]	inhibition to IMPDH2	IC_{50} = 12.64±1.86μM	*Penicillium bialowiezense*
metabolites 8-O-methyl mycophenolic acid (**370**)	[141]	inhibition to IMPDH2	IC_{50} = 0.92±0.08μM	*Penicillium bialowiezense*
3-hydroxymycophenolic acid (**371**)	[141]	inhibition to IMPDH2	IC_{50} = 0.95±0.09μM	*Penicillium bialowiezense*
N-mycophenoyl-*L*-valine (**374**)	[141]	inhibition to IMPDH2	IC_{50} = 15.73±1.65μM	*Penicillium bialowiezense*
N-mycophenoyl-*L*-phenyloalanine (**373**)	[141]	inhibition to IMPDH2	IC_{50} = 23.76±3.54μM	*Penicillium bialowiezense*
N-mycophenoyl-*L*-alanine (**374**)	[141]	inhibition to IMPDH2	IC_{50} = 17.52±1.30μM	*Penicillium bialowiezense*
melilotigenin C (**395**)	[94]	inhibition to pancreatic lipase	IC_{50} = 15.6 μg/mL	*Aspergillus* sp.
pseurotin A (**409**)	[99]	increased the number of osteoclasts	0.1 μM	*Pseudallescheria boydii*
pseurotin A (**409**)	[99-102]	inhibited the production of IgE	IC_{50} =3.6 μM	*Pseudallescheria boydii*
AM6898B (**410**)	[99]	increased the number of osteoclasts	both 0.1 and 1.0 μM	*Pseudallescheria boydii*
(−)-ovalicin derivative (**411**)	[99]	increased the number of osteoclasts	both 0.1 and 1.0 μM	*Pseudallescheria boydii*
chlovalicin (**412**)	[99]	decreased the number of osteoclasts	both 0.1 and 1.0 μM	*Pseudallescheria boydii*
penicillide (**414**)	[51-54]	calcium-activated papain-like protease	IC_{50} = 7.1 mu M	*Penicillium pinophilum*
aluminiumneoaspergillin (**419**) *	[19]	lethality against brine shrimp	LC_{50} = 6.61 μM	*Aspergillus* sp.
zirconiumneoaspergillin (**420**) *	[19]	lethality against brine shrimp	LC_{50} =10.76 μM	*Aspergillus* sp.
ferrineoaspergillin (**421**)	[19]	lethality against brine shrimp	LC_{50} = 29.62 μM	*Aspergillus* sp.

Table S6. Distribution of the compounds according to chemical structure.

Type	Sub-type				Total	Percentage
Terpenes	Sesquiterpenes (17)	Diterpenoids (8)	Triterpenes (1)	Meroterpenoids (10)	36	8.51%
Alkaloids	Diketopiperazines (33)	Quinazolinone alkaloids (24)	Cytochalasins (15)	Other alkaloids (31)	103	24.35%
Peptides and Depsipeptides	Cyclopeptides (25)		Linear peptides (21)		46	10.87%
Aromatics	Polyketides (44)	Anthraquinone (37)	Dimmers (11)	Other Aromatic Compounds (58)	150	35.46%
Lactones	49				49	11.58%
Steroids	16				16	3.79%
Other compounds	23				23	5.44%
Total					423	100%

Table S7. The strain source of the natural products from coral-derived fungi.

	Terpenes	Alkaloids	Peptides and Depsipeptides	Aromatics	Lactones	Steroids	Other compounds	Total	Percentage
Soft coral	28	51	17	81	25	11	11	224	52.96%
Gorgonian-derived	6	39	28	46	21	4	12	156	36.88%
Hard coral	2	1	1	5	1			10	2.36%
leather coral	-	7	-	1	-	-	-	8	1.89%
Scleractinia stony coral	-	3	-	17	-	-	-	20	4.73%
Unknown diseased coral	-	2	-	-	2	1	-	5	1.18%
Total	36	103	46	150	49	16	23	423	100%

References

[1] H.-J. Li, W.-J. Lan, C.-K. Lam, F. Yang, X.-F. Zhu, Hirsutane Sesquiterpenoids from the Marine-Derived Fungus Chondrostereum sp, Chemistry & Biodiversity 8(2) (2011) 317-324.

[2] H.-J. Li, T. Chen, Y.-L. Xie, W.-D. Chen, X.-F. Zhu, W.-J. Lan, Isolation and Structural Elucidation of Chondrosterins F-H from the Marine Fungus Chondrostereum sp, Marine Drugs 11(2) (2013) 551-558.

[3] H.-J. Li, Y.-L. Xie, Z.-L. Xie, Y. Chen, C.-K. Lam, W.-J. Lan, Chondrosterins A-E, Triquinane-Type Sesquiterpenoids from Soft Coral-Associated Fungus Chondrostereum sp, Marine Drugs 10(3) (2012) 627-638.

[4] R. Riga, N. Happyana, E. Holisotan Hakim, Sesquiterpenes produced by Pestalotiopsis microspora HF 12440 isolated from Artocarpus heterophyllus, Nat Prod Res 34(15) (2020) 2229-2231.

[5] Z.Y. Guo, M.H. Tan, C.X. Liu, M.M. Lv, Z.S. Deng, F. Cao, K. Zou, P. Proksch, Aspergoterpenins A(-)D: Four New Antimicrobial Bisabolane Sesquiterpenoid Derivatives from an Endophytic Fungus Aspergillus versicolor, Molecules 23(6) (2018).

[6] I. Wijesekara, Y.-X. Li, T.-S. Vo, Q. Van Ta, D.-H. Ngo, S.-K. Kim, Induction of apoptosis in human cervical carcinoma HeLa cells by neoechinulin A from marine-derived fungus Microsporum sp, Process Biochemistry 48(1) (2013) 68-72.

[7] X. Wei, C. Feng, S.-Y. Wang, D.-M. Zhang, X.-H. Li, C.-X. Zhang, New Indole Diketopiperazine Alkaloids from Soft Coral-Associated Epiphytic Fungus Aspergillus sp. EGF 15-0-3, Chemistry & Biodiversity 17(5) (2020).

[8] B.N.S. Ningsih, V. Rukachaisirikul, S. Phongpaichit, S. Preedanon, J. Sakayaroj, C. Muanprasat, A nonadride derivative from the marine-derived fungus Aspergillus chevalieri PSU-AMF79, Natural Product Research 8.

[9] L. Hu, T. Zhang, D. Liu, G. Guan, J. Huang, P. Proksch, X. Chen, W. Lin, Notoamide-type alkaloid induced apoptosis and autophagy via a P38/JNK signaling pathway in hepatocellular carcinoma cells, Rsc Advances 9(34) (2019) 19855-19868.

[10] Z. Cheng, D. Liu, W. Cheng, P. Proksch, W. Lin, Versiquinazolines L-Q, new polycyclic alkaloids from the marine-derived fungus Aspergillus versicolor, Rsc Advances 8(55) (2018) 31427-31439.

[11] X.-W. Luo, C.-H. Gao, H.-M. Lu, J.-M. Wang, Z.-Q. Su, H.-M. Tao, X.-F. Zhou, B. Yang, Y.-H. Liu, HPLC-DAD-Guided Isolation of Diversified Chaetoglobosins from the Coral-Associated Fungus Chaetomium globosum C2F17, Molecules 25(5) (2020).

[12] Q.C. Zheng, M.Z. Kong, Q. Zhao, G.D. Chen, H.Y. Tian, X.X. Li, L.D. Guo, J. Li, Y.Z. Zheng, H. Gao, Chaetoglobosin Y, a new cytochalasan from Chaetomium globosum, Fitoterapia 93 (2014) 126-31.

[13] H. Dou, Y.X. Song, X.Q. Liu, W. Gong, E.G. Li, R.X. Tan, Y.Y. Hou, Chaetoglobosin Fex from the Marine-Derived Endophytic Fungus Inhibits Induction of Inflammatory Mediators via Toll-Like Receptor 4 Signaling in Macrophages, Biol. Pharm. Bull. 34(12) (2011) 1864-1873.

[14] C.-J. Zheng, C.-L. Shao, L.-Y. Wu, M. Chen, K.-L. Wang, D.-L. Zhao, X.-P. Sun, G.-Y. Chen, C.-Y. Wang, Bioactive Phenylalanine Derivatives and Cytochalasins from the Soft Coral-Derived Fungus, Aspergillus elegans, Marine Drugs 11(6) (2013) 2054-2068.

[15] Y. Erden, S. Tekin, K. Betul Ceylan, C. Tekin, S. Kirbag, Antioxidant, Antimicrobial and Anticancer Activities of the Aspergillin PZ and Terphenyllin Secondary Metabolites: An in vitro Study, Gazi University Journal of Science 32(3) (2019) 792-800.

[16] N. Naruse, H. Yamamoto, S. Murata, Y. Sawada, Y. Fukagawa, T. Oki, ASPOCHALASIN-E, A NEW ANTIBIOTIC ISOLATED FROM A FUNGUS, Journal of Antibiotics 46(4) (1993) 679-681.

[17] J.-d. Hao, J.-j. Zheng, M. Chen, C.-y. Wang, Cytochalasins from the Gorgonian-Derived Fungus Aspergillus sp. XS-2009-0B15, Chemistry of Natural Compounds 53(4) (2017) 732-735.

[18] L.-H. Huang, Y.-X. Chen, J.-C. Yu, J. Yuan, H.-J. Li, W.-Z. Ma, R. Watanapokasin, K.-C. Hu, S.I. Niaz, D.-P. Yang, W.-J. Lan, Secondary Metabolites from the Marine-Derived Fungus Dichotomomyces sp. L-8 and Their Cytotoxic Activity, Molecules 22(3) (2017).

[19] X. Xu, F. He, X. Zhang, J. Bao, S. Qi, New mycotoxins from marine-derived fungus Aspergillus sp. SCSGAF0093, Food Chem Toxicol 53 (2013) 46-51.

[20] F. Zhu, J.S. Wu, G.Y. Chen, W.H. Lu, J.H. Pan, Biosynthesis, Characterization and Biological Evalutation of Fe(III) and Cu(II) Complexes of Neoaspergillic Acid, a Hydroxamate Siderophore Produced by Co-cultures of two Marine-derived Mangrove Epiphytic Fungi, Natural Product Communications 6(8) (2011) 1137-1140.

[21] M.S. Elnaggar, S.S. Ebada, M.L. Ashour, W. Ebrahim, W.E.G. Mueller, A. Mandi, T. Kurtan, A. Singab, W. Lin, Z. Liu, P. Proksch, Xanthones and sesquiterpene derivatives from a marine-derived fungus Scopulariopsis sp, Tetrahedron 72(19) (2016) 2411-2419.

[22] X.M. Hou, Y.H. Zhang, Y. Hai, J.Y. Zheng, Y.C. Gu, C.Y. Wang, C.L. Shao, Aspersymmetide A, a New Centrosymmetric Cyclohexapeptide from the Marine-Derived Fungus Aspergillus versicolor, Mar Drugs 15(11) (2017).

[23] M. Zhu, H. Gao, C. Wu, T. Zhu, Q. Che, Q. Gu, P. Guo, D. Li, Lipid-lowering polyketides from a soft coral-derived fungus Cladosporium sp TZP29, Bioorganic & Medicinal Chemistry Letters 25(17) (2015) 3606-3609.

[24] X.M. Hou, C.Y. Wang, Y.C. Gu, C.L. Shao, Penimethavone A, a flavone from a gorgonian-derived fungus Penicillium chrysogenum, Nat Prod Res 30(20) (2016) 2274-7.

[25] C.-N. Wang, H.-M. Lu, C.-H. Gao, L. Guo, Z.-Y. Zhan, J.-J. Wang, Y.-H. Liu, S.-T. Xiang, J. Wang, X.-W. Luo, Cytotoxic benzopyranone and xanthone derivatives from a coral symbiotic fungusCladosporium halotoleransGXIMD 02502, Natural Product Research (2020).

[26] M.W. Xia, C.B. Cui, C.W. Li, C.J. Wu, J.X. Peng, D.H. Li, Rare Chromones from a Fungal Mutant of the Marine-Derived Penicillium purpurogenum G59, Mar Drugs 13(8) (2015) 5219-36.

[27] J. Li, Y.-B. Zheng, T. Kurtan, M.-X. Liu, H. Tang, C.-L. Zhuang, W. Zhang, Anthraquinone derivatives from a coral associated fungusStemphylium lycopersici, Natural Product Research 34(15) (2020) 2116-2123.

[28] Y.R. Lin, K.C. Peng, M.H. Chan, H.L. Peng, S.Y. Liu, Effect of Pachybasin on General Toxicity and Developmental Toxicity in Vivo, J Agric Food Chem 65(48) (2017) 10489-10494.
[29] M. Deng, Y. Xue, L. Xu, Q. Wang, J. Wei, X. Ke, J. Wang, X. Chen, Chrysophanol exhibits inhibitory activities against colorectal cancer by targeting decorin, Cell Biochem Funct 38(1) (2020) 47-57.
[30] T. Shi, X.-M. Hou, Z.-Y. Li, F. Cao, Y.-H. Zhang, J.-Y. Yu, D.-L. Zhao, C.-L. Shao, C.-Y. Wang, Harzianumnones A and B: two hydroxyanthraquinones from the coral-derived fungus Trichoderma harzianum, Rsc Advances 8(49) (2018) 27596-27601.
[31] R.P. Maskey, I. Grun-Wollny, H. Laatsch, Isolation, structure elucidation and biological activity of 8-O-methylaverufin and 1,8-O-dimethylaverantin as new antifungal agents from Penicillium chrysogenum, Journal of Antibiotics 56(5) (2003) 459-463.
[32] K. Sakai, S. Ohte, T. Ohshiro, D. Matsuda, R. Masuma, L.L. Rudel, H. Tomoda, Selective inhibition of acyl-CoA : cholesterol acyltransferase 2 isozyme by flavasperone and sterigmatocystin from Aspergillus species, Journal of Antibiotics 61(9) (2008) 568-572.
[33] M. Chen, C.L. Shao, C.J. Kong, Z.G. She, C.Y. Wang, A NEW ANTHRAQUINONE DERIVATIVE FROM A GORGONIAN-DERIVED FUNGUS Aspergillus sp, Chemistry of Natural Compounds 50(4) (2014) 617-620.
[34] R. Tang, A. Kimishima, A. Setiawan, M. Arai, Secalonic acid D as a selective cytotoxic substance on the cancer cells adapted to nutrient starvation, J Nat Med 74(2) (2020) 495-500.
[35] H. Ren, L. Tian, Q.Q. Gu, W.M. Zhu, Secalonic acid D; A cytotoxic constituent from marine lichen-derived fungus Gliocladium sp T31, Archives of Pharmacal Research 29(1) (2006) 59-63.
[36] J. Wang, X.H. Nong, X.Y. Zhang, X.Y. Xu, M. Amin, S.H. Qi, Screening of Anti-Biofilm Compounds from Marine-Derived Fungi and the Effects of Secalonic Acid D on Staphylococcus aureus Biofilm, J Microbiol Biotechnol 27(6) (2017) 1078-1089.
[37] C.L. Shao, C.Y. Wang, M.Y. Wei, Y.C. Gu, Z.G. She, P.Y. Qian, Y.C. Lin, Aspergilones A and B, two benzylazaphilones with an unprecedented carbon skeleton from the gorgonian-derived fungus Aspergillus sp, Bioorg Med Chem Lett 21(2) (2011) 690-3.
[38] P. Fu, F. Kong, Y. Wang, Y. Wang, P. Liu, G. Zuo, W. Zhu, Antibiotic Metabolites from the Coral-Associated Actinomycete Streptomyces sp OUCMDZ-1703, Chinese Journal of Chemistry 31(1) (2013) 100-104.
[39] D.-L. Zhao, F. Cao, C.-Y. Wang, L.-J. Yang, T. Shi, K.-L. Wang, C.-L. Shao, C.-Y. Wang, Alternatone A, an Unusual Perylenequinone-Related Compound from a Soft-Coral-Derived Strain of the Fungus Alternaria alternata, Journal of Natural Products 82(11) (2019) 3201-3204.
[40] X. Zhang, X.X. Liu, Y.N. Xing, M. Zhang, Y. Zhao, Y.Y. Wei, B. Zhang, R.H. Jiao, Alternatones A and B, two polyketides possessing novel skeletons from entophyte Alternaria alternate L-10, J Asian Nat Prod Res 24(4) (2022) 353-360.
[41] M. Wang, L. Yang, L. Feng, F. Hu, F. Zhang, J. Ren, Y. Qiu, Z. Wang, Verruculosins A-B, New Oligophenalenone Dimers from the Soft Coral-Derived Fungus Talaromyces verruculosus, Marine Drugs 17(9) (2019).
[42] G. Said, X.-M. Hou, X. Liu, R. Chao, Y.-Y. Jiang, J.-Y. Zheng, C.-L. Shao, Antimicrobial and Cytotoxic Activities of Secondary Metabolites from the Soft Coral Derived Fungus Aspergillus sp, Chemistry of Natural Compounds 55(3) (2019) 531-533.
[43] M. Cheng, P. Li, Y. Jiang, X. Tang, W. Zhang, Q. Wang, G. Li, Penitol A and Penicitols E-I: Citrinin Derivatives from Penicillium citrinum and the Structure Revision of Previously Proposed Analogues, Journal of Natural Products 84(4) (2021) 1345-1352.
[44] M. Chen, L. Han, C.L. Shao, Z.G. She, C.Y. Wang, Bioactive Diphenyl Ether Derivatives from a Gorgonian-Derived Fungus Talaromyces sp, Chemistry & Biodiversity 12(3) (2015) 443-450.
[45] G. Said, X.-F. Mou, Y.-W. Fang, T.-M. Liang, M.-Y. Wei, G.-Y. Chen, C.-L. Shao, Secondary Metabolites Isolated from the Soft Coral-Derived Fungus Aspergillus sp from the South China Sea, Chemistry of Natural Compounds 54(3) (2018) 547-549.
[46] M. Liu, W. Sun, J. Wang, Y. He, J. Zhang, F. Li, C. Qi, H. Zhu, Y. Xue, Z. Hu, Y. Zhang, Bioactive secondary metabolites from the marine-associated fungus Aspergillus terreus, Bioorganic Chemistry 80 (2018) 525-530.
[47] B. Liu, N. Chen, Y. Xu, J.W. Zhang, Y. Sun, L.Z. Zhao, Y.B. Ji, A new benzophenone with biological activities from metabolites of butyrolactone I in rat faeces, Nat Prod Res 35(15) (2021) 2489-2497.
[48] Y. Nie, J. Yang, L. Zhou, Z. Yang, J. Liang, Y. Liu, X. Ma, Z. Qian, P. Hong, A.V. Kalueff, C. Song, Y. Zhang, Marine fungal metabolite butyrolactone I prevents cognitive deficits by relieving inflammation and intestinal microbiota imbalance on aluminum trichloride-injured zebrafish, J Neuroinflammation 19(1) (2022) 39.
[49] B. Yang, J. Long, X. Pang, X. Lin, S. Liao, J. Wang, X. Zhou, Y. Li, Y. Liu, Structurally diverse polyketides and phenylspirodrimanes from the soft coral-associated fungus Stachybotrys chartarum SCSIO41201, Journal of Antibiotics 74(3) (2021) 190-198.
[50] J.-Y. Long, J.-F. Wang, S.-R. Liao, X.-P. Lin, X.-F. Zhou, Y.-Q. Li, B. Yang, Y.-H. Liu, Four new steroids from the marine soft coral-derived fungus Penicillium sp. SCSIO41201, Chinese Journal of Natural Medicines 18(4) (2020) 250-255.
[51] D.L. Zhao, C.L. Shao, Q. Zhang, K.L. Wang, F.F. Guan, T. Shi, C.Y. Wang, Azaphilone and Diphenyl Ether Derivatives from a Gorgonian-Derived Strain of the Fungus Penicillium pinophilum, J Nat Prod 78(9) (2015) 2310-4.
[52] M.C. Chung, H.J. Lee, H.K. Chun, Y.H. Kho, Penicillide, a nonpeptide calpain inhibitor, produced by Penicillium sp. F60760, J. Microbiol. Biotechnol. 8(2) (1998) 188-190.
[53] C. Yimnual, S. Satitsri, B.N.S. Ningsih, V. Rukachaisirikul, C. Muanprasat, A fungus-derived purpactin A as an inhibitor of TMEM16A chloride channels and mucin secretion in airway epithelial cells, Biomed Pharmacother 139 (2021) 111583.
[54] A.A. Sy-Cordero, M. Figueroa, H.A. Raja, M.E. Meza Avina, M.P. Croatt, A.F. Adcock, D.J. Kroll, M.C. Wani, C.J. Pearce, N.H. Oberlies, Spiroscytalin, a new tetramic acid and other metabolites of mixed biogenesis from Scytalidium cuboideum, Tetrahedron 71(47) (2015) 8899-904.
[55] F. Cao, Q. Yang, C.L. Shao, C.J. Kong, J.J. Zheng, Y.F. Liu, C.Y. Wang, Bioactive 7-Oxabicyclic[6.3.0]lactam and 12-Membered Macrolides from a Gorgonian-Derived Cladosporium sp. Fungus, Mar Drugs 13(7) (2015) 4171-8.

[56] C.-Y. Wang, Y.-F. Liu, F. Cao, C.-Y. Wang, Bisabolane-Type Sesquiterpenoids from a Gorgonian-Derived Aspergillus sp. Fungus Induced by DNA Methyltransferase Inhibitor, Chemistry of Natural Compounds 52(6) (2016) 1129-1132.

[57] M.-Y. Wei, C.-Y. Wang, Q.-A. Liu, C.-L. Shao, Z.-G. She, Y.-C. Lin, Five Sesquiterpenoids from a Marine-Derived Fungus Aspergillus sp Isolated from a Gorgonian Dichotella gemmacea, Marine Drugs 8(4) (2010) 941-949.

[58] X.D. Li, X.M. Li, G.M. Xu, P. Zhang, B.G. Wang, Antimicrobial Phenolic Bisabolanes and Related Derivatives from Penicillium aculeatum SD-321, a Deep Sea Sediment-Derived Fungus, J Nat Prod 78(4) (2015) 844-9.

[59] C. Zheng, C. Shao, K. Wang, D. Zhao, Y. Wang, C. Wang, Secondary metabolites and their bioactivities of a soft coral-derived fungus Aspergillus versicolor(ZJ-2008015), Chinese Journal of Marine Drugs 31(2) (2012) 7-13.

[60] C.-J. Zheng, C.-L. Shao, M. Chen, Z.-G. Niu, D.-L. Zhao, C.-Y. Wang, Merosesquiterpenoids and Ten-Membered Macrolides from a Soft Coral-Derived Lophiostoma sp Fungus, Chemistry & Biodiversity 12(9) (2015) 1407-1414.

[61] Y. Dai, Y. Lin, X. Pang, X. Luo, L. Salendra, J. Wang, X. Zhou, Y. Lu, B. Yang, Y. Liu, Peptides from the Soft Coral-associated Fungus Simplicillium sp SCSIO41209, Phytochemistry 154 (2018) 56-62.

[62] C.-L. Shao, R.-F. Xu, M.-Y. Wei, Z.-G. She, C.-Y. Wang, Structure and Absolute Configuration of Fumiquinazoline L, an Alkaloid from a Gorgonian-Derived Scopulariopsis sp Fungus, Journal of Natural Products 76(4) (2013) 779-782.

[63] Y. Zhuang, X. Teng, Y. Wang, P. Liu, G. Li, W. Zhu, New Quinazolinone Alkaloids within Rare Amino Acid Residue from Coral-Associated Fungus, Aspergillus versicolor LCJ-5-4, Organic Letters 13(5) (2011) 1130-1133.

[64] Z.-L. Guo, J.-J. Zheng, F. Cao, C. Wang, C.-Y. Wang, Chemical Constituents of the Gorgonian-Derived Fungus Chaetomium globosum, Chemistry of Natural Compounds 53(1) (2017) 199-202.

[65] S.S. Ebada, W. Ebrahim, A new antibacterial quinolone derivative from the endophytic fungus Aspergillus versicolor strain Eich.5.2.2, South African Journal of Botany 134 (2020) 151-155.

[66] C.-L. Shao, R.-F. Xu, C.-Y. Wang, P.-Y. Qian, K.-L. Wang, M.-Y. Wei, Potent Antifouling Marine Dihydroquinolin-2(1H)-one-Containing Alkaloids from the Gorgonian Coral-Derived Fungus Scopulariopsis sp, Marine Biotechnology 17(4) (2015) 408-415.

[67] M. Liu, Y. He, L. Shen, W.H. Al Anbari, H. Li, J. Wang, C. Qi, Z. Hu, Y. Zhang, Asperteramide A, an Unusual N-Phenyl-Carbamic Acid Methyl Ester Trimer Isolated from the Coral-Derived Fungus Aspergillus Terreus, European Journal of Organic Chemistry 2019(18) (2019) 2928-2932.

[68] Seven naphtho-γ-pyrones from the marine-derived fungus Alternaria alternata: structure elucidation and biological properties, Organic & Medicinal Chemistry Letters 2(1) (2012) 6.

[69] M. Chen, C.L. Shao, H. Meng, Z.G. She, C.Y. Wang, Anti-respiratory syncytial virus prenylated dihydroquinolone derivatives from the gorgonian-derived fungus Aspergillus sp. XS-20090B15, J Nat Prod 77(12) (2014) 2720-4.

[70] M. Chen, C.L. Shao, X.M. Fu, C.J. Kong, Z.G. She, C.Y. Wang, Lumazine peptides penilumamides B-D and the cyclic pentapeptide asperpeptide A from a gorgonian-derived Aspergillus sp. fungus, J Nat Prod 77(7) (2014) 1601-6.

[71] Y.-L. Jia, M.-Y. Wei, H.-Y. Chen, F.-F. Guan, C.-Y. Wang, C.-L. Shao, (+)- and ()-Pestaloxazine A, a Pair of Antiviral Enantiomeric Alkaloid Dimers with a Symmetric Spiro oxazinane-piperazinedione Skeleton from Pestalotiopsis sp, Organic Letters 17(17) (2015) 4216-4219.

[72] X. Ma, X.-H. Nong, Z. Ren, J. Wang, X. Liang, L. Wang, S.-H. Qi, Antiviral peptides from marine gorgonian-derived fungus Aspergillus sp. SCSIO 41501, Tetrahedron Letters 58(12) (2017) 1151-1155.

[73] A.P. Michael, E.J. Grace, M. Kotiw, R.A. Barrow, Isochromophilone IX, a novel GABA-containing metabolite isolated from a cultured fungus, Penicillium sp, Aust. J. Chem. 56(1) (2003) 13-15.

[74] N.-N. Wu, X.-M. Hou, M.-Y. Wei, J.-Y. Zheng, C.-L. Shao, Antifungal and Antibacterial Activities of Azaphilones from the Gorgonian-Derived Penicillium sclerotiorum Fungus, Chemistry of Natural Compounds 55(3) (2019) 549-551.

[75] M.Y. Wei, C.F. Wang, K.L. Wang, P.Y. Qian, C.Y. Wang, C.L. Shao, Preparation, Structure, and Potent Antifouling Activity of Sclerotioramine Derivatives, Mar Biotechnol (NY) 19(4) (2017) 372-378.

[76] Y.F. Liu, Y.H. Zhang, C.L. Shao, F. Cao, C.Y. Wang, Microketides A and B, Polyketides from a Gorgonian-Derived Microsphaeropsis sp. Fungus, J Nat Prod 83(4) (2020) 1300-1304.

[77] C.-J. Zheng, C.-L. Shao, Z.-Y. Guo, J.-F. Chen, D.-S. Deng, K.-L. Yang, Y.-Y. Chen, X.-M. Fu, Z.-G. She, Y.-C. Lin, C.-Y. Wang, Bioactive Hydroanthraquinones and Anthraquinone Dimers from a Soft Coral-Derived Alternaria sp Fungus, Journal of Natural Products 75(2) (2012) 189-197.

[78] M. Shaaban, K.A. Shaaban, M.S. Abdel-Aziz, Seven naphtho-gamma-pyrones from the marine-derived fungus Alternaria alternata: structure elucidation and biological properties, Organic and medicinal chemistry letters 2 (2012) 6-6.

[79] J. Bao, Y.-L. Sun, X.-Y. Zhang, Z. Han, H.-C. Gao, F. He, P.-Y. Qian, S.-H. Qi, Antifouling and antibacterial polyketides from marine gorgonian coral-associated fungus Penicillium sp SCSGAF 0023, Journal of Antibiotics 66(4) (2013) 219-223.

[80] M.-Y. Wei, D. Li, C.-L. Shao, D.-S. Deng, C.-Y. Wang, (+/-)-Pestalachloride D, an Antibacterial Racemate of Chlorinated Benzophenone Derivative from a Soft Coral-Derived Fungus Pestalotiopsis sp, Marine Drugs 11(4) (2013) 1050-1060.

[81] C.-F. Wang, Y. Wang, X.-L. Zhang, M.-Y. Wei, C.-Y. Wang, C.-L. Shao, Two Dichlorinated Benzophenone Derivatives from the Soft Coral-Derived Pestalotiopsis sp Fungus and Their Antibacterial Activity, Chemistry of Natural Compounds 53(6) (2017) 1174-1176.

[82] N.R. Pace, A molecular view of microbial diversity and the biosphere, Science 276(5313) (1997) 734-740.

[83] C.S. Anyanwu, J.L. Sorensen, Secondary Metabolites from a Strain of Alternaria tenuissima Isolated from Northern Manitoba Soil, Natural Product Communications 10(1) (2015) 39-42.

[84] X. Zhu, Y. Chen, X. Tang, D. Wang, Y. Miao, J. Zhang, R. Li, L. Zhang, J. Chen, General toxicity and genotoxicity of altertoxin I: A novel 28-day multiendpoint assessment in male Sprague-Dawley rats, J Appl Toxicol (2022).
[85] Y. Zang, G. Genta-Jouve, A.E. Escargueil, A.K. Larsen, L. Guedon, B. Nay, S. Prado, Antimicrobial Oligophenalenone Dimers from the Soil Fungus Talaromyces stipitatus, J Nat Prod 79(12) (2016) 2991-2996.
[86] L. Wang, Y. Huang, L. Zhang, Z. Liu, W. Liu, H. Xu, Q. Zhang, H. Zhang, Y. Yan, Z. Liu, T. Zhang, W. Zhang, C. Zhang, Structures and absolute configurations of phomalones from the coral-associated fungus Parengyodontium album sp. SCSIO 40430, Organic & Biomolecular Chemistry 19(27) (2021) 6030-6037.
[87] J.-x. Li, X.-x. Lei, Y.-h. Tan, Y.-h. Liu, B. Yang, Y.-q. Li, Two new bioactive polyphenols from the soft coral-derived fungus Talaromyces sp. SCSIO 041201, Natural Product Research (2020).
[88] T.-M. Liang, Y.-W. Fang, J.-Y. Zheng, C.-L. Shao, Secondary Metabolites Isolated from the Gorgonian-Derived Fungus Aspergillus ruber and Their Antiviral Activity, Chemistry of Natural Compounds 54(3) (2018) 559-561.
[89] Y. Li, X. Li, U. Lee, J.S. Kang, H.D. Choi, B.W. Son, A new radical scavenging anthracene glycoside, asperflavin ribofuranoside, and polyketides from a marine isolate of the fungus Microsporum, Chemical & Pharmaceutical Bulletin 54(6) (2006) 882-883.
[90] T. Shi, J. Qi, C.L. Shao, D.L. Zhao, X.M. Hou, C.Y. Wang, Bioactive Diphenyl Ethers and Isocoumarin Derivatives from a Gorgonian-Derived Fungus Phoma sp. (TA07-1), Mar Drugs 15(6) (2017).
[91] K. Nuankeaw, B. Chaiyosang, T. Suebrasri, S. Kanokmedhakul, S. Lumyong, S. Boonlue, First report of secondary metabolites, Violaceol I and Violaceol II produced by endophytic fungus, Trichoderma polyalthiae and their antimicrobial activity, Mycoscience 61(1) (2020) 16-21.
[92] Y. Asami, J.H. Jang, H. Oh, J.H. Sohn, J.W. Kim, D.O. Moon, O. Kwon, M. Kawatani, H. Osada, B.Y. Kim, J.S. Ahn, Violaceols Function as Actin Inhibitors Inducing Cell Shape Elongation in Fibroblast Cells, Biosci. Biotechnol. Biochem. 76(8) (2012) 1431-1437.
[93] M. Chen, K.L. Wang, M. Liu, Z.G. She, C.Y. Wang, Bioactive Steroid Derivatives and Butyrolactone Derivatives from a Gorgonian-Derived Aspergillus sp Fungus, Chemistry & Biodiversity 12(9) (2015) 1398-1406.
[94] Q. Peng, J. Cai, J. Long, B. Yang, X. Lin, J. Wang, J. Xiao, Y. Liu, X. Zhou, New azaphthalide and phthalide derivatives from the marine coral-derived fungus Aspergillus sp. SCSIO41405, Phytochemistry Letters 43 (2021) 94-97.
[95] D.-W. Sun, F. Cao, M. Liu, F.-F. Guan, C.-Y. Wang, New Fatty Acid From a Gorgonian-Derived Xylaria sp. Fungus, Chemistry of Natural Compounds 53(2) (2017) 227-230.
[96] M.L. Yu, F.F. Guan, F. Cao, Y.L. Jia, C.Y. Wang, A new antiviral pregnane from a gorgonian-derived Cladosporium sp. fungus, Nat Prod Res 32(11) (2018) 1260-1266.
[97] J.-Y. Yu, T. Shi, Y. Zhou, Y. Xu, D.-L. Zhao, C.-Y. Wang, Naphthalene derivatives and halogenate quinoline from the coral-derived fungus Trichoderma harzianum (XS-20090075) through OSMAC approach, Journal of Asian Natural Products Research 23(3) (2021) 250-257.
[98] G. Li, S. Kusari, C. Golz, H. Laatsch, C. Strohmann, M. Spiteller, Epigenetic Modulation of Endophytic Eupenicillium sp. LG41 by a Histone Deacetylase Inhibitor for Production of Decalin-Containing Compounds, J Nat Prod 80(4) (2017) 983-988.
[99] D.-H. Liu, Y.-Z. Sun, T. Kurtan, A. Mandi, H. Tang, J. Li, L. Su, C.-L. Zhuang, Z.-Y. Liu, W. Zhang, Osteoclastogenesis Regulation Metabolites from the Coral-Associated Fungus Pseudallescheria boydii TW-1024-3, Journal of Natural Products 82(5) (2019) 1274-1282.
[100] M.A.U. Mehedi, A.H. Molla, P. Khondkar, S. Sultana, M.A. Islam, M.A. Rashid, R. Chowdhury, Pseurotin A: An Antibacterial Secondary Metabolite from Aspergillus fumigatus, Asian J. Chem. 22(4) (2010) 2611-2614.
[101] G. Schmeda-Hirschmann, E. Hormazabal, J.A. Rodriguez, C. Theoduloz, Cycloaspeptide A and pseurotin A from the endophytic fungus Penicillium janczewskii, Z.Naturforsch.(C) 63(5-6) (2008) 383-388.
[102] M. Ishikawa, T. Ninomiya, H. Akabane, N. Kushida, G. Tsujiuchi, M. Ohyama, S. Gomi, K. Shito, T. Murata, Pseurotin A and its analogues as inhibitors of immunoglobuline E production, Bioorganic & Medicinal Chemistry Letters 19(5) (2009) 1457-1460.
[103] Z. Liu, P. Qiu, H. Liu, J. Li, C. Shao, T. Yan, W. Cao, Z. She, Identification of anti-inflammatory polyketides from the coral-derived fungus Penicillium sclerotiorin: In vitro approaches and molecular-modeling, Bioorganic Chemistry 88 (2019).
[104] M.H. Teiten, F. Mack, A. Debbab, A.H. Aly, M. Dicato, P. Proksch, M. Diederich, Anticancer effect of altersolanol A, a metabolite produced by the endophytic fungus Stemphylium globuliferum, mediated by its pro-apoptotic and anti-invasive potential via the inhibition of NF-kappaB activity, Bioorg Med Chem 21(13) (2013) 3850-8.
[105] Z. Liu, G. Xia, S. Chen, Y. Liu, H. Li, Z. She, Eurothiocin A and B, Sulfur-Containing Benzofurans from a Soft Coral-Derived Fungus Eurotium rubrum SH-823, Marine Drugs 12(6) (2014) 3669-3680.
[106] H.J. Shin, C.V. Anh, D.Y. Cho, D.K. Choi, J.S. Kang, P.T.H. Trinh, B.K. Choi, H.S. Lee, New Polyenes from the Marine-Derived Fungus Talaromyces cyanescens with Anti-Neuroinflammatory and Cytotoxic Activities, Molecules 26(4) (2021).
[107] M. Liu, Q. Zhou, J. Wang, J. Liu, C. Qi, Y. Lai, H. Zhu, Y. Xue, Z. Hu, Y. Zhang, Anti-inflammatory butenolide derivatives from the coral-derived fungus Aspergillus terreus and structure revisions of aspernolides D and G, butyrolactone VI and 4,8-diacetoxy butyrolactone VI, Rsc Advances 8(23) (2018) 13040-13047.
[108] J. Li, H. Tao, X.-x. Lei, H. Zhang, X. Zhou, Y. Liu, Y. Li, B. Yang, Arthriniumsteroids A-D, four new steroids from the soft coral-derived fungus Simplicillium lanosoniveum SCSIO41212, Steroids 171 (2021).
[109] X.-P. Sun, Y. Xu, F. Cao, R.-F. Xu, X.-L. Zhang, C.-Y. Wang, Isoechinulin-Type Alkaloids from a Soft Coral-Derived Fungus Nigrospora oryzae, Chemistry of Natural Compounds 50(6) (2014) 1153-1155.

[110] W.-J. Lan, K.-T. Wang, M.-Y. Xu, J.-J. Zhang, C.-K. Lam, G.-H. Zhong, J. Xu, D.-P. Yang, H.-J. Li, L.-Y. Wang, Secondary metabolites with chemical diversity from the marine-derived fungus Pseudallescheria boydii F19-1 and their cytotoxic activity, Rsc Advances 6(80) (2016) 76206-76213.
[111] X.-F. Mou, X. Liu, R.-F. Xu, M.-Y. Wei, Y.-W. Fang, C.-L. Shao, Scopuquinolone B, a new monoterpenoid dihydroquinolin-2(1H)-one isolated from the coral-derived Scopulariopsis sp fungus, Natural Product Research 32(7) (2018) 773-776.
[112] K.A.U. Zaman, J.H. Park, L. DeVine, Z. Hu, X. Wu, H.S. Kim, S. Cao, Secondary Metabolites from the Leather Coral-Derived Fungal Strain Xylaria sp. FM1005 and Their Glycoprotein IIb/IIIa Inhibitory Activity, Journal of Natural Products 84(2) (2021) 466-473.
[113] C.-J. Zheng, X.-M. Fu, X.-L. Zhang, W.-W. Kong, C.-Y. Wang, Bioactive Perylene Derivatives from a Soft Coral-Derived Fungus Alternaria sp. (ZJ-2008017), Chemistry of Natural Compounds 51(4) (2015) 766-768.
[114] M. Matsuoka, B. Wispriyono, Y. Iryo, H. Igisu, T. Sugiura, Inhibition of HgCl2-induced mitogen-activated protein kinase activation by LL-Z1640-2 in CCRF-CEM cells, Eur. J. Pharmacol. 409(2) (2000) 155-158.
[115] C.L. Shao, H.X. Wu, C.Y. Wang, Q.A. Liu, Y. Xu, M.Y. Wei, P.Y. Qian, Y.C. Gu, C.J. Zheng, Z.G. She, Y.C. Lin, Potent antifouling resorcylic acid lactones from the gorgonian-derived fungus Cochliobolus lunatus, J Nat Prod 74(4) (2011) 629-33.
[116] S.M. Hande, J.i. Uenishi, Total synthesis of aspergillide B and structural discrepancy of aspergillide A, Tetrahedron Letters 50(2) (2009) 189-192.
[117] T. Nagasawa, S. Kuwahara, Enantioselective synthesis of aspergillide B, Biosci Biotechnol Biochem 73(8) (2009) 1893-4.
[118] J. Bao, X.-Y. Zhang, X.-Y. Xu, F. He, X.-H. Nong, S.-H. Qi, New cyclic tetrapeptides and asteltoxins from gorgonian-derived fungus Aspergillus sp. SCSGAF 0076, Tetrahedron 69(9) (2013) 2113-2117.
[119] M. Chen, C.-L. Shao, K.-L. Wang, Y. Xu, Z.-G. She, C.-Y. Wang, Dihydroisocoumarin derivatives with antifouling activities from a gorgonian-derived Eurotium sp. fungus, Tetrahedron 70(47) (2014) 9132-9138.
[120] N. Bunbamrung, C. Intaraudom, A. Dramae, N. Boonyuen, S. Veeranondha, P. Rachtawee, P. Pittayakhajonwut, Antimicrobial activity of illudalane and alliacane sesquiterpenes from the mushroom Gloeostereum incarnatum BCC41461, Phytochemistry Letters 20 (2017) 274-281.
[121] Z. Xiong, X. Cao, Q. Wen, Z. Chen, Z. Cheng, X. Huang, Y. Zhang, C. Long, Y. Zhang, Z. Huang, An overview of the bioactivity of monacolin K / lovastatin, Food Chem Toxicol 131 (2019) 110585.
[122] D.-L. Zhao, L.-J. Yang, T. Shi, C.-Y. Wang, C.-L. Shao, C.-Y. Wang, Potent Phytotoxic Harziane Diterpenes from a Soft Coral-Derived Strain of the Fungus Trichoderma harzianum XS-20090075, Scientific Reports 9 (2019).
[123] C. Zhao, C. Zhang, F. He, W. Zhang, A. Leng, X. Ying, Two new alkaloids from Portulaca oleracea L. and their bioactivities, Fitoterapia 136 (2019) 104166.
[124] W.L. Wang, Z.Y. Lu, H.W. Tao, T.J. Zhu, Y.C. Fang, Q.Q. Gu, W.M. Zhu, Isoechinulin-type alkaloids, variecolorins A-L, from halotolerant Aspergillus variecolor, Journal of Natural Products 70(10) (2007) 1558-1564.
[125] H. Fujimoto, T. Fujimaki, E. Okuyama, M. Yamazaki, Immunomodulatory constituents from an ascomycete, Microascus tardifaciens, Chemical & Pharmaceutical Bulletin 47(10) (1999) 1426-1432.
[126] O.I. Zhuravleva, A.S. Antonov, V.T.D. Trang, M.V. Pivkin, Y.V. Khudyakova, V.A. Denisenko, R.S. Popov, N.Y. Kim, E.A. Yurchenko, A.V. Gerasimenko, A.A. Udovenko, G.v. Amsberg, S.A. Dyshlovoy, S.S. Afiyatullov, New Deoxyisoaustamide Derivatives from the Coral-Derived Fungus Penicillium dimorphosporum KMM 4689, Marine Drugs 19(1) (2021).
[127] J.E. Wulff, S.B. Herzon, R. Siegrist, A.G. Myers, Evidence for the rapid conversion of stephacidin B into the electrophilic monomer avrainvillamide in cell culture, J. Am. Chem. Soc. 129(16) (2007) 4898-+.
[128] R. Mikkola, M.A. Andersson, M. Hautaniemi, M.S. Salkinoja-Salonen, Toxic indole alkaloids avrainvillamide and stephacidin B produced by a biocide tolerant indoor mold Aspergillus westerdijkiae, Toxicon 99 (2015) 58-67.
[129] Z. Cheng, L. Lou, D. Liu, X. Li, P. Proksch, S. Yin, W. Lin, Versiquinazolines A-K, Fumiquinazoline-Type Alkaloids from the Gorgonian-Derived Fungus Aspergillus versicolor LZD-14-1, J Nat Prod 79(11) (2016) 2941-2952.
[130] S.J. Choo, B.S. Yun, I.J. Ryoo, Y.H. Kim, K.H. Bae, I.D. Yoo, Aspochalasin I, a Melanogenesis Inhibitor from Aspergillus sp, J. Microbiol. Biotechnol. 19(4) (2009) 368-371.
[131] C.Y. An, X.M. Li, H. Luo, C.S. Li, M.H. Wang, G.M. Xu, B.G. Wang, 4-Phenyl-3,4-dihydroquinolone derivatives from Aspergillus nidulans MA-143, an endophytic fungus isolated from the mangrove plant Rhizophora stylosa, J Nat Prod 76(10) (2013) 1896-901.
[132] X.-M. Hou, Y.-Y. Li, Y.-W. Shi, Y.-W. Fang, R. Chao, Y.-C. Gu, C.-Y. Wang, C.-L. Shao, Integrating Molecular Networking and H-1 NMR To Target the Isolation of Chrysogeamides from a Library of Marine-Derived Penicillium Fungi, Journal of Organic Chemistry 84(3) (2019) 1228-1237.
[133] R. Chen, Z. Cheng, J. Huang, D. Liu, C. Wu, P. Guo, W. Lin, Versicotides D–F, new cyclopeptides with lipid-lowering activities, RSC Adv. 7(78) (2017) 49235-49243.
[134] P. Battilani, A. Pietri, Ochratoxin A in grapes and wine, Eur. J. Plant Pathol. 108(7) (2002) 639-643.
[135] M.P. Lopez-Gresa, M.C. Gonzalez, J. Primo, P. Moya, V. Romero, E. Estornell, Circumdatin H, a new inhibitor of mitochondrial NADH oxidase, from Aspergillus ochraceus, Journal of Antibiotics 58(6) (2005) 416-419.
[136] Y.-Z. Sun, T. Kurtan, A. Mandi, H. Tang, Y. Chou, K. Soong, L. Su, P. Sun, C.-L. Zhuang, W. Zhang, Immunomodulatory Polyketides from a Phoma-like Fungus Isolated from a Soft Coral, Journal of Natural Products (2017).
[137] S. Tsukamoto, S. Miura, Y. Yamashita, T. Ohta, Aspermytin A: a new neurotrophic polyketide isolated from a marine-derived fungus of the genus Aspergillus, Bioorg Med Chem Lett 14(2) (2004) 417-20.

[138] H. Zhang, X.-X. Lei, S. Shao, X. Zhou, Y. Li, B. Yang, Azaphilones and Meroterpenoids from the Soft Coral-Derived Fungus Penicillium glabrum glmu003, Chemistry & Biodiversity (2021).
[139] M. Wang, M. Sun, H. Hao, C. Lu, Avertoxins A-D, Prenyl Asteltoxin Derivatives from Aspergillus versicolor Y10, an Endophytic Fungus of Huperzia serrata, J Nat Prod 78(12) (2015) 3067-70.
[140] J. Ma, X.-L. Zhang, Y. Wang, J.-Y. Zheng, C.-Y. Wang, C.-L. Shao, Aspergivones A and B, two new flavones isolated from a gorgonian-derived Aspergillus candidus fungus, Natural Product Research 31(1) (2017) 32-36.
[141] Q. Zhang, B. Yang, F. Li, M. Liu, S. Lin, J. Wang, Y. Xue, H. Zhu, W. Sun, Z. Hu, Y. Zhang, Mycophenolic Acid Derivatives with Immunosuppressive Activity from the Coral-Derived Fungus Penicillium bialowiezense, Marine Drugs 16(7) (2018).
